# Development and validation of glycosyltransferase related-gene for the diagnosis and prognosis of head and neck squamous cell carcinoma

**DOI:** 10.18632/aging.205455

**Published:** 2024-01-19

**Authors:** Miao He, Li Wang, Zihan Yue, Chunbo Feng, Guosheng Dai, Jinsong Jiang, Hui Huang, Qingjun Ji, Minglang Zhou, Dapeng Li, Wei Chai

**Affiliations:** 1 Department of Otorhinolaryngology, Head and Neck Surgery, The People’s Hospital of Bozhou, Bozhou 236000, Anhui, China; 2 Scientific Research and Experiment Center, The People’s Hospital of Bozhou, Bozhou 236000, Anhui, China; 3 Second Clinical College, Tongji Medical College, Huazhong University of Science and Technology, Wuhan 430000, Hubei, China

**Keywords:** glycosyltransferases, prognosis, diagnosis, signature, head and neck squamous cell carcinoma

## Abstract

Background: Head and neck squamous cell carcinoma (HNSCC) is a highly heterogeneous cancer characterized by difficulties in early diagnosis and outcome prediction. Aberrant glycosylated structures produced by the aberrant expression of glycosyltransferases are prevalent in HNSCC. In this study, we aim to construct glycosyltransferase-related gene signatures with diagnostic and prognostic value to better stratify patients with HNSCC and improve their diagnosis and prognosis.

Methods: Bioinformatic tools were used to process data of patients with HNSCC from The Cancer Genome Atlas (TCGA) database. The prognostic model was formatted using univariate and multivariate Cox regression methods, while the diagnostic signature was constructed using support vector machine (SVM) and LASSO analysis. The results were verified using the Gene Expression Omnibus (GEO) cohort. The tumor microenvironment and benefits of immune checkpoint inhibitor (ICI) therapy in subgroups defined by glycosyltransferase-related genes were analyzed. Molecular biology experiments, including western blotting, cell counting kit (CCK)-8, colony formation, wound healing, and Transwell assays, were conducted to confirm the oncogenic function of beta-1,4-galactosyltransferase 3 (B4GALT3) in HNSCC.

Results: We established a five-gene prognostic signature and a 15-gene diagnostic model. Based on the median risk score, patients with low risk had longer overall survival than those in the high-risk group, which was consistent with the results of the GEO cohort. The concrete results suggested that high-risk samples were related to a high tumor protein (TP)53 mutation rate, high infiltration of resting memory cluster of differentiation (CD)4 T cells, resting natural killer (NK) cells, and M0 macrophages, and benefited from ICI therapy. In contrast, the low-risk subgroup was associated with a low TP53 mutation rate; and high infiltration of naive B cells, plasma cells, CD8 T cells, and resting mast cells; and benefited less from ICI therapy. In addition, the diagnostic model had an area under curve (AUC) value of 0.997 and 0.978 in the training dataset and validation cohort, respectively, indicating the high diagnostic potential of the model. Ultimately, the depletion of B4GALT3 significantly hindered the proliferation, migration, and invasion of HNSCC cells.

Conclusions: We established two new biomarkers that could provide clinicians with diagnostic, prognostic, and treatment guidance for patients with HNSCC.

## INTRODUCTION

Head and neck squamous cell carcinomas (HNSCC) occur in the mucosal epithelium of the oral cavity, pharynx, and larynx and are the most common malignancies of the head and neck [1]. The incidence of HNSCC is increasing and is anticipated to increase by 30% by 2030 [1, 2]. According to the clinical stage, HNSCC is normally treated with surgical resection, followed by adjuvant radiotherapy or chemoradiotherapy. Despite the substantial development of combined-modality therapy, the five-year survival rate of patients with HNSCC has been lower than 50%, primarily due to the difficulty in early diagnosis, significant molecular heterogeneity between tumors, and resistance to chemotherapy and radiotherapy [3]. Therefore, early diagnosis and appropriate stratification of patients with HNSCC may be key to aiding individualized treatment decisions and improving survival rates.

Glycosylation, a series of reactions that produce complicated carbohydrate structures (glycans) attached to the backbone of a polypeptide, is a major posttranslational modification in cellular development [4]. This process primarily involves the sequential action of different families of glycosylases, such as glycosyltransferases and glycosidases, whose expression and function are tightly regulated in each cell [5]. Tumor cells typically express high levels of characteristic glycan structures, and differential changes in the cellular glycan structure are primarily associated with altered expression patterns of glycosyltransferase genes [6]. There is evidence that alterations in the glycan structure may play a significant role in the classification and subtypes of malignancies. Deep modification of glycosyltransferase gene expression produces aberrant glycosylation and deregulates the glycosylation of cancer cells, leading to an aggressive phenotype. This is a sign of tumor cell metamorphosis [7–10].

Various glycoproteins have been identified as potential therapeutic targets for HNSCC. Sialic acid, an abnormally glycosylated mucin with significantly elevated levels in patients with HNSCC, is strongly associated with tumor progression and metastasis [11–13]. Bergler et al. indicated that the epidermal growth factor receptor, which carries a sialoglycan structure in the extracellular region, is highly expressed in HNSCC [14]. Previous findings have suggested that the expression of epidermal growth factors may be associated with more aggressive and metastatic tumors [15]. Hence, sialic acid bound to the surface of cancer cells may be a useful target for anticancer therapy [16]. Mucin 1 (MUC1) is a transmembrane glycoprotein that has been proven to be a probable biomarker for predicting the prognosis of invasive HNSCC [17]. MUC1 enhanced double-strand-break repair and resistance to ionizing radiation (IR)-induced apoptosis in HNSCC cells. Radiation resistance and sensitivity have also been observed in HNSCC cells with high and low MUC1 expression, respectively [18]. Therefore, MUC1 may be a potential target for improving the efficacy of integrated treatments in patients with HNSCC.

In summary, the relevance of glycoproteins to HNSCC cell invasion and metastasis cannot be ignored. In this study, we stratified patients with HNSCC according to glycosyltransferase gene expression profiles involved in glycoconjugate biosynthesis. To optimize the therapeutic system for HNSCC, we constructed a glycosyltransferase-related diagnostic and prognostic signature using bioinformatics. The diagnostic and prognostic values of these glycosyltransferase genes have been confirmed in public databases, providing novel possible markers for clinical diagnosis and treatment decisions in patients with HNSCC.

## MATERIALS AND METHODS

### Public datasets collection

RNA-seq data and relevant clinical characteristics of patients with HNSCC from The Cancer Genome Atlas (TCGA) database were downloaded as the training cohort. Simultaneously, we used data from GSE41613 as the test cohort for the prognostic model and the matrix files of GSE127165 as validation data for the diagnostic model. The GSE41613 and GSE127165 datasets contained clinical information and gene expression profiles of patients with HNSCC obtained from the Gene Expression Omnibus (GEO) database. We identified 169 glycosyltransferase-related genes in a study by Mohamed Abd-El-Halim et al. [19] and downloaded them for further analysis (Supplementary Table 1). All the data used in our study are publicly available.

### Identification and validation of prognostic gene signature

In the TCGA cohort, the R package “limma” was used to recognize the differentially expressed genes (DEGs) by comparing the different gene expression levels of tumor and normal cells. The thresholds were set as |log_2_FC| > 0.5 along with a false discovery rate < 0.05. Univariate Cox analysis was performed on these DEGs, and valuable genes (P<0.05) were further analyzed using multivariate analysis. The risk score was calculated using the following formula: Risk score = expression level of gene-1 × coefficient of gene-1 + expression level of gene-2 × coefficient of gene-2 +. . .+ expression level of gene-n × coefficient of gene-n [20]. Glycosyltransferase-related genes screened using the aforementioned methods were used to build a prognostic signature of patients with HNSCC. The expression levels of each gene were multiplied by the corresponding Cox coefficient and added to calculate the risk score for all clinical cases. HNSCC samples were then segmented into high- and low-risk subgroups using the median risk score. The prognostic power of the model was evaluated using Kaplan-Meier (K-M) survival curves with time-dependent receiver operating characteristic (ROC) curves. The same process was completed for the GSE41613 dataset to validate the signature stability. Finally, to estimate the independent prognostic value of the glycosyltransferase-related gene signature, univariate and multivariate Cox regression analyses were performed.

### Functional enrichment analysis and mutation gene analysis

To better comprehend the biological functions of the target genes, the “clusterProfiler” package was used to perform gene set enrichment analysis (GSEA) of the two subgroups based on the Kyoto Encyclopedia of Genes and Genomes. In addition, somatic mutation information was collected from the cBioPortal database and gene mutation analysis was performed on both subgroups using the “Maftools” package.

### Evaluation of immune cell infiltration and immunotherapy

To explore the correlation between the glycosyltransferase-related gene model and immune cell levels, the CIBERSORT (https://cibersort.stanford.edu/) algorithm was used to handle the expression data of each case to assess the proportion of different immune cells in the two risk groups of the TCGA cohort [21]. In addition, we analyzed the association of the prognostic risk score with immune checkpoint (IC) expression levels and immunotherapy scores.

### Construction and evaluation of diagnostic model

In the TCGA cohort, two machine learning algorithms, the least absolute shrinkage and selection operator (LASSO) and support vector machine (SVM), were used to select genes with diagnostic value. SVM is a machine-learning technique widely used for tumor gene expression profile analysis and tumor marker detection in different types of cancers [22]. LASSO regression can reduce the number of estimated parameters while maintaining a high prediction accuracy, thereby reducing data overfitting and making the model easy to visualize and interpret [23]. The intersection genes of the two algorithms were used to construct predictive diagnostic models. ROC curve was used to examine the effectiveness of the model. As with the prognostic model, we used matrix data from GEO for model validation.

### Cell culture

FaDu cells were obtained from the American Type Culture Collection (USA), and TU177 cells were procured from Otwo Biotech, Inc. (Shenzhen, China). These cells were cultured in a 37° C incubator with a 5% CO_2_ atmosphere using DMEM or RPMI-1640 medium supplemented with 1% penicillin-streptomycin and 10% fetal bovine serum.

### Lentiviral infection

TU177 cells were stably transduced with lentiviral vectors (GV248, GenePharma, Shanghai, China) carrying specific short hairpin RNAs (shRNAs) targeting B4GALT3. The shRNA sequences used were as follows: shB4GALT3-1, 5’-GGGATGAACTCACTGACATAC-3’, shB4GALT3-2, 5’-GGACGCAAGATGGGATGAACT-3’, and control scrambled shRNA (shSc), 5’-TTCTCCGAACGTGTCACGT-3’. Cell lines overexpressing B4GALT3 were established using the lentiviral vector GV492 containing the complete B4GALT3 sequence (GenePharma).

### Western blot analysis

Immunoblotting was performed according to standard procedures [24]. Briefly, cell lysates were prepared using NuPAGE 4-12% Bis-Tris gels and subsequently transferred onto PVDF membranes (Millipore, USA). Before the application of primary antibodies, the membranes were blocked with 5% nonfat milk solution to prevent nonspecific binding of antibodies. Following overnight incubation at 4° C with primary antibodies and subsequent one-hour incubation with secondary antibodies, chemiluminescence was used to visualize the results. The commercial antibody anti-B4GALT3 (11041-1-AP, Proteintech, China) was used in these experiments.

### *In vitro* functional assays

*In vitro* functional assessments, including CCK-8 assay (TargetMol; Shanghai, China), colony formation, wound healing, and Transwell assays, were employed to assess the functional roles of B4GALT3.

### Statistical analysis

R software and various packages were used for the statistical analyses. Univariate and multivariate Cox regression analyses were used to screen the prognostic genes and independent prognostic parameters. Survival analysis was performed using the K-M analysis with the log-rank test. Spearman’s correlation analysis was performed to analyze the relationships. The Wilcoxon test was used for comparison between subgroups.

### Data availability statement

Publicly available datasets were analyzed in this study. The raw data of this study are derived from the TCGA database (https://portal.gdc.cancer.gov/) and the GEO data portal (https://www.ncbi.nlm.nih.gov/geo/), which are publicly available databases.

## RESULTS

### Glycosyltransferase-related DEGs were identified

**Figure 1:**
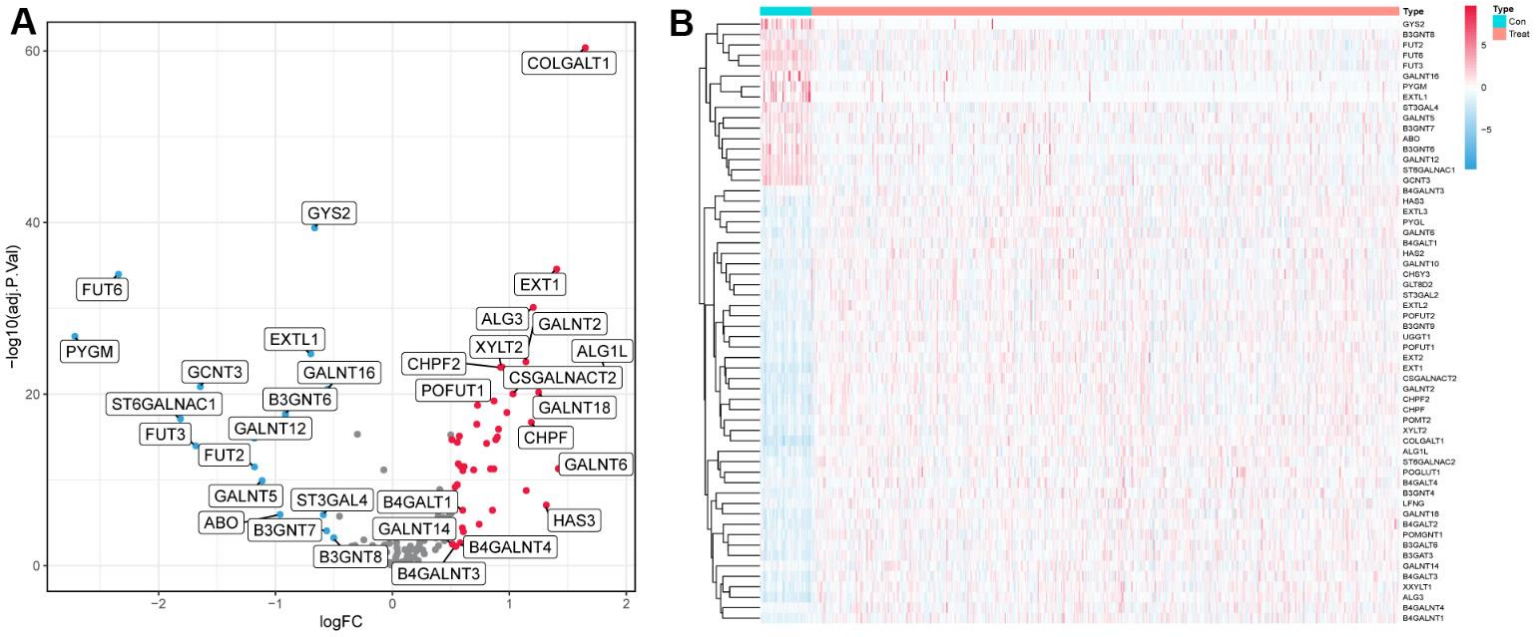
**Differentially expressed glycosyltransferase-related genes.** (**A**) Volcano map of differential genes, the red nodes represent upregulated genes while the blue nodes represent downregulated genes. (**B**) Heatmaps showing differentially expressed genes in the TCGA dataset. The color indicates the level of expression of the gene (red represents upregulation, blue represents downregulation).

In the TCGA cohort, we analyzed the differential expression of 169 glycosyltransferase-related genes in 502 tumors and 44 normal tissues and obtained a total of 58 DEGs (Figure 1A), of which 42 were upregulated and 16 were downregulated (Figure 1B).

### Establishment and validation of the glycosyltransferase-related gene prognostic signature

Univariate Cox analysis was performed on the candidate genes obtained in the above process, and a total of 12 genes exhibited statistically significant differences (*P<0.05*) (Supplementary Figure 1). To further identify independent prognostic genes in patients with HNSCC, multivariate Cox analysis was performed on 12 glycosyltransferase-related genes. As shown in Table 1, five significant genes (B4GALT3, PYGL, GALNT14, FUT2, and GALNT16) were screened to construct a prognostic signature. The risk score of all tumor samples was counted as follows: Risk score = (0.974* B4GALT3) + (0.418* PYGL) + (0.195* GALNT14) + (−0.413* FUT2) + (−0.886* GALNT16).

**Table 1:** Five genes identified by multivariate Cox regression analysis used to construct prognostic models in the TCGA cohort.

**Gene**	**Coefficient**	**HR**	**95%CI**	**P-value**
B4GALT3	0.974	2.647	1.003-6.988	0.049
PYGL	0.418	1.519	1.085-2.128	0.015
GALNT14	0.195	1.216	0.953-1.550	0.115
FUT2	-0.431	0.650	0.511-0.828	<0.001
GALNT16	-0.866	0.421	0.193-0.915	0.029

**Figure 2:**
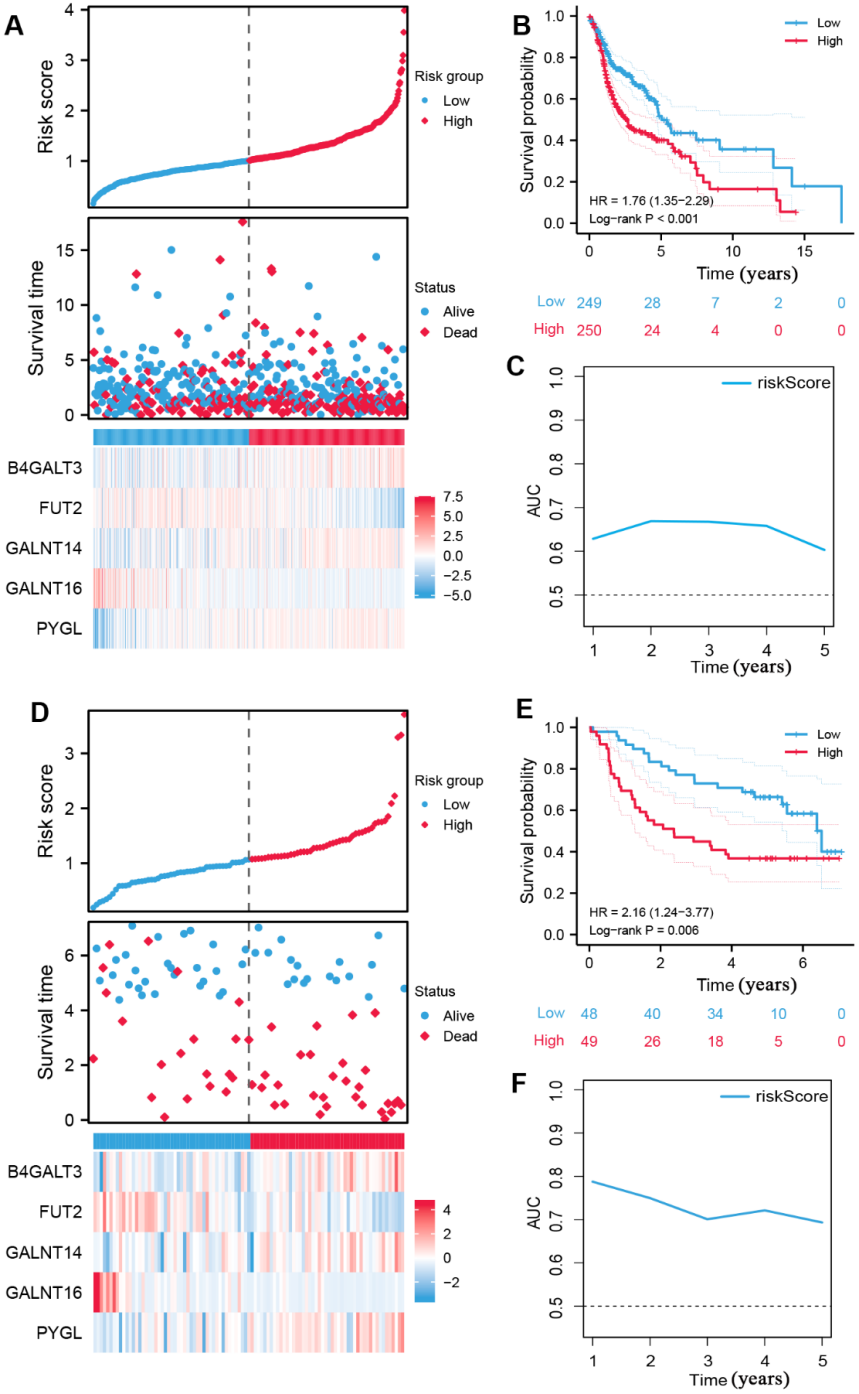
**Construction of prognostic signature of five glycosyltransferase-related genes.** (**A**–**C**) The prognostic signature was constructed in the TCGA dataset. (**A**) The distribution of risk score, OS status, and the heatmap of the expression profiles of signature genes, (**B**) K-M survival curves based on the prognostic signature, and (**C**) the AUCs of glycosyltransfer related gene signature. (**D**–**F**) Evaluate the performance of the prognostic signature in the GSE41613 dataset. (**D**) The distribution of risk score, OS status, and the heatmap of the expression profiles of signature genes, (**E**) K-M survival curves based on the prognostic signature, and (**F**) the AUCs of glycosyltransfer related gene signature.

Using the median risk score as the cut-off value, all patients with HNSCC were classified into high- and low-risk subgroups (Figure 2A), and high-risk samples exhibited poorer overall survival (OS) in comparison to low-risk samples (log-rank P < 0.001, Figure 2B). ROC curves were used to assess the capability of the signature and the AUC at one, three, and five years in Figure 2C shows moderate sensitivity and specificity of the prognostic signature. Additionally, validation was performed using the GSE41613 dataset. The same formula was used to compute the risk score, and the 97 patients were classified into two subgroups based on the median risk score (Figure 2D). The K-M curve and AUC of the ROC curves showed significance similar to that of the TCGA cohort (Figure 2E, 2F). These results demonstrate the reliability and stability of the prognostic signature.

### Independent analysis of prognostic signature

**Figure 3:**
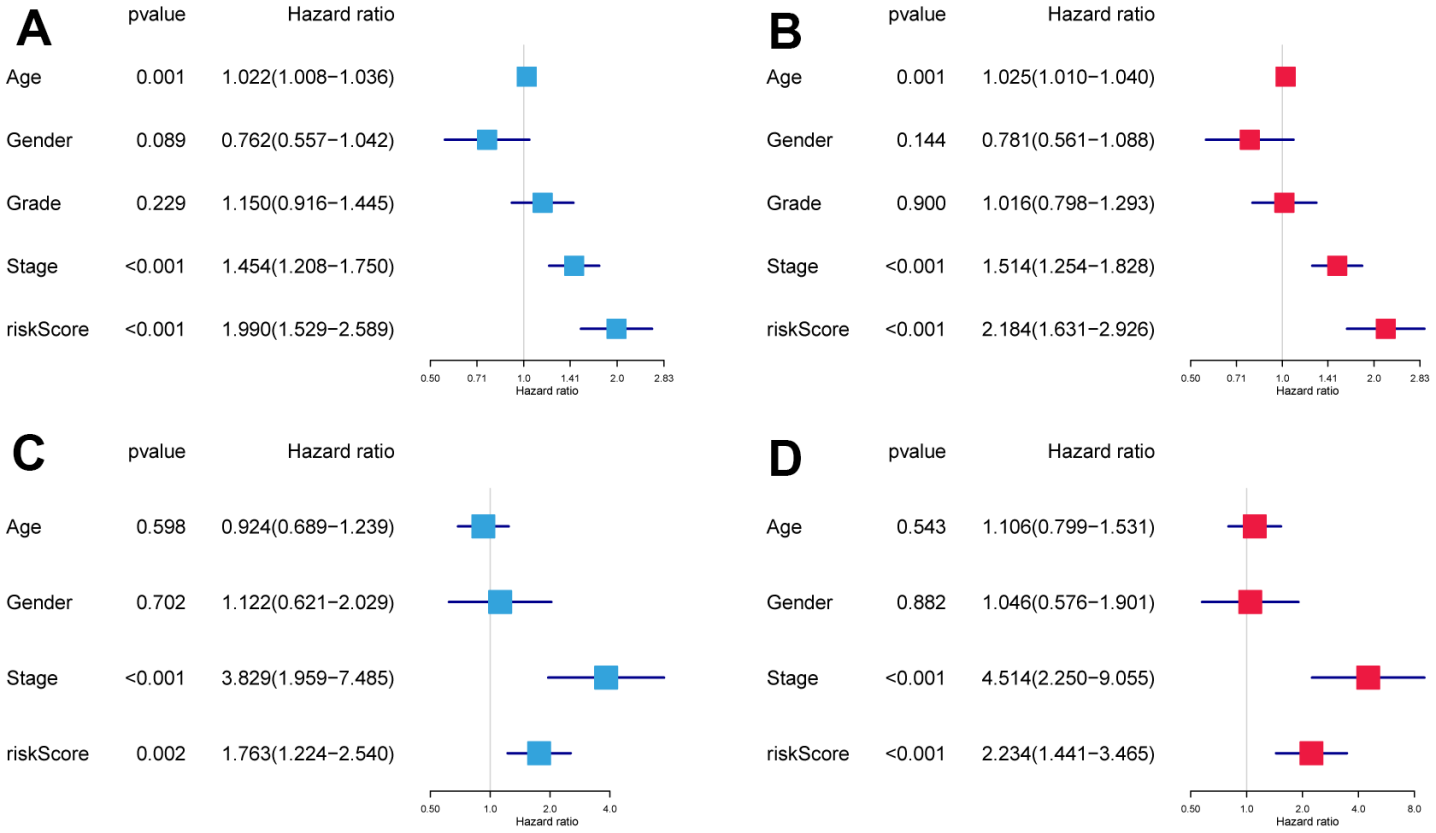
**The independence identification of the risk model.** Univariate and multivariate Cox regression analysis has been performed in the TCGA cohort (**A**, **B**) and GSE41613 cohort (**C**, **D**).

Risk scores and other clinicopathological characteristics (age, sex, grade, and stage) were combined for independent analyses. Univariate and multivariate Cox analysis suggested that risk score was a significantly independent prognostic factor for OS (TCGA cohort univariate: hazard ratio (HR) = 1.990, 95% confidence interval (CI) = 1.529−2.589, P < 0.001, Figure 3A; TCGA cohort multivariate: HR = 2.184, 95% CI = 1.631−2.926, P < 0.001, Figure 3B). This was confirmed again in the GSE41613 validation cohort (Figure 3C, 3D).

### GSEA analysis and mutant landscape

**Figure 4:**
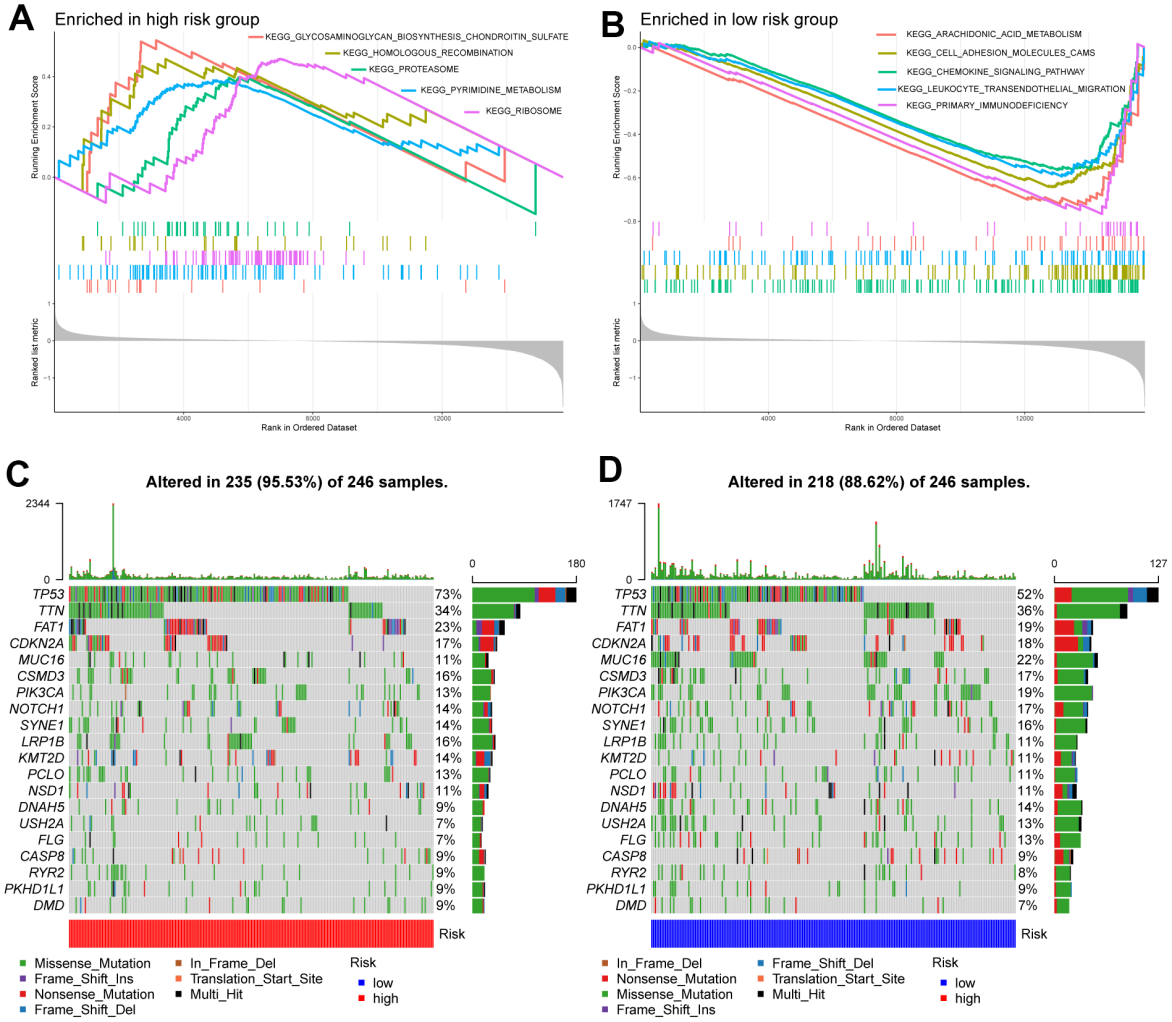
**Molecular characteristics of high- and low-risk groups.** (**A**) Gene sets enriched in the high-risk group. (**B**) Gene sets enriched in the low-risk group. The mutation profile of the top 20 mutation genes in high-risk patients (**C**) and low-risk patients (**D**).

The results of GSEA revealed that the high-risk subgroup samples were usually enriched in transcription and protein regulation-related pathways, such as glycosaminoglycan biosynthesis of chondroitin sulfate, proteasomes, and ribosomes (Figure 4A). Groups with low risk tended to activate arachidonic acid metabolism, cell adhesion molecules (CAMs), chemokine signaling pathways, and primary immunodeficiency pathways, among others (Figure 4B).

Next, we performed gene mutation analysis according to the glycosyltransferase-related gene signature and observed that the mutation count in the high subgroup was higher than that in the low subgroup. Moreover, the top 20 mutated genes in the high- (Figure 4C), and low-risk (Figure 4D) samples were contradistinguished. Additionally, we observed that missense variations were the most common mutation type, and all samples were dominated by mutations in TP53, titin (TTN), and FAT atypical cadherin 1 (FAT1) in both groups.

### The associations with immune microenvironment and ICI therapy

**Figure 5:**
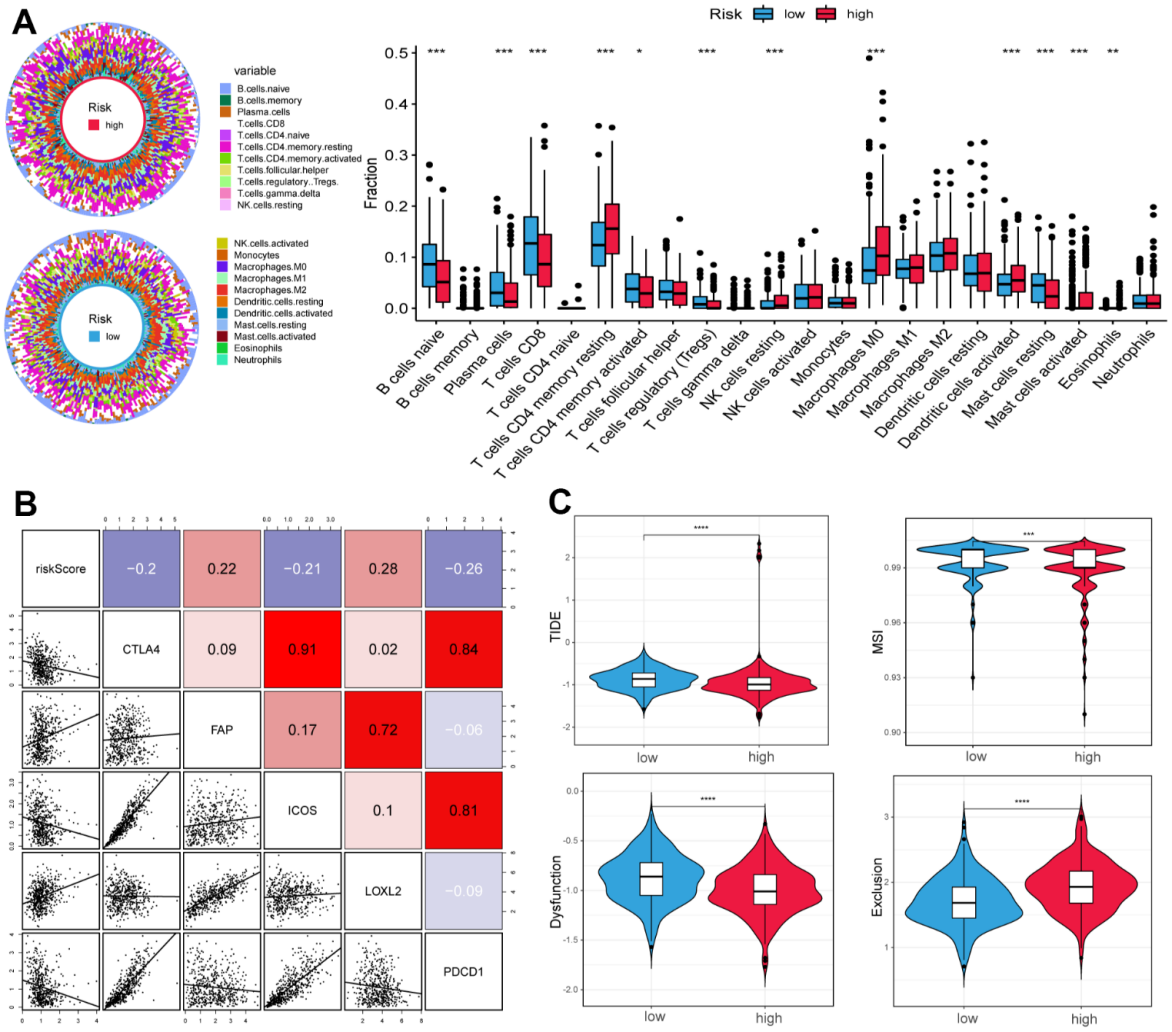
**Correlation of tumor microenvironment (TME) landscape and ICI therapy with risk score.** (**A**) The proportions of TME cells in high- and low-risk groups. Significant statistical differences between the two subgroups were assessed using the Wilcoxon test. (**B**) Linear regression among immune checkpoints (CTLA4, FAP, ICOS, LOXL2, and PDCD1) and risk scores, and the numbers placed on the right of the plot represent coefficients. (**C**) TIDE, MSI, and T cell exclusion and dysfunction score in two groups. (*p < 0.05, ** p < 0.01, *** p < 0.001, **** p < 0.0001).

To further explore the differences in immune cells between the two subgroups, the CIBERSORT method was used to quantify the immune infiltration scores, and the Wilcoxon test was performed for comparison. We found that low-risk samples were enriched in naive B cells, plasma cells, CD8 T cells, regulatory T cells (Tregs), and resting mast cells, whereas resting memory CD4 T cells, resting NK cells, M0 macrophages, and activated dendritic cells were more abundant in high-risk samples (Figure 5A). We then calculated the Spearman correlation coefficient between the risk score and common immune checkpoint**,** the outcomes suggested that the risk score was positively correlated to fibroblast activation protein (FAP) and lysyl oxidase-like 2 (LOXL2), and negatively correlated with cytotoxic T lymphocyte-associated protein 4 (CTLA4), ICOS, and programmed cell death protein 1 (PDCD1) (Figure 5B).

In addition, we used the tumor immune dysfunction and exclusion (TIDE) and microsatellite instability (MSI) scores to evaluate the potential value of immunotherapy. In our study, the high-risk group had lower TIDE and MSI scores, which suggested that this group may benefit significantly from immunotherapy than the low-risk group (Figure 5C). Finally, the low-risk group had lower T cell exclusion scores and higher T cell dysfunction.

### Diagnostic model construction

**Figure 6:**
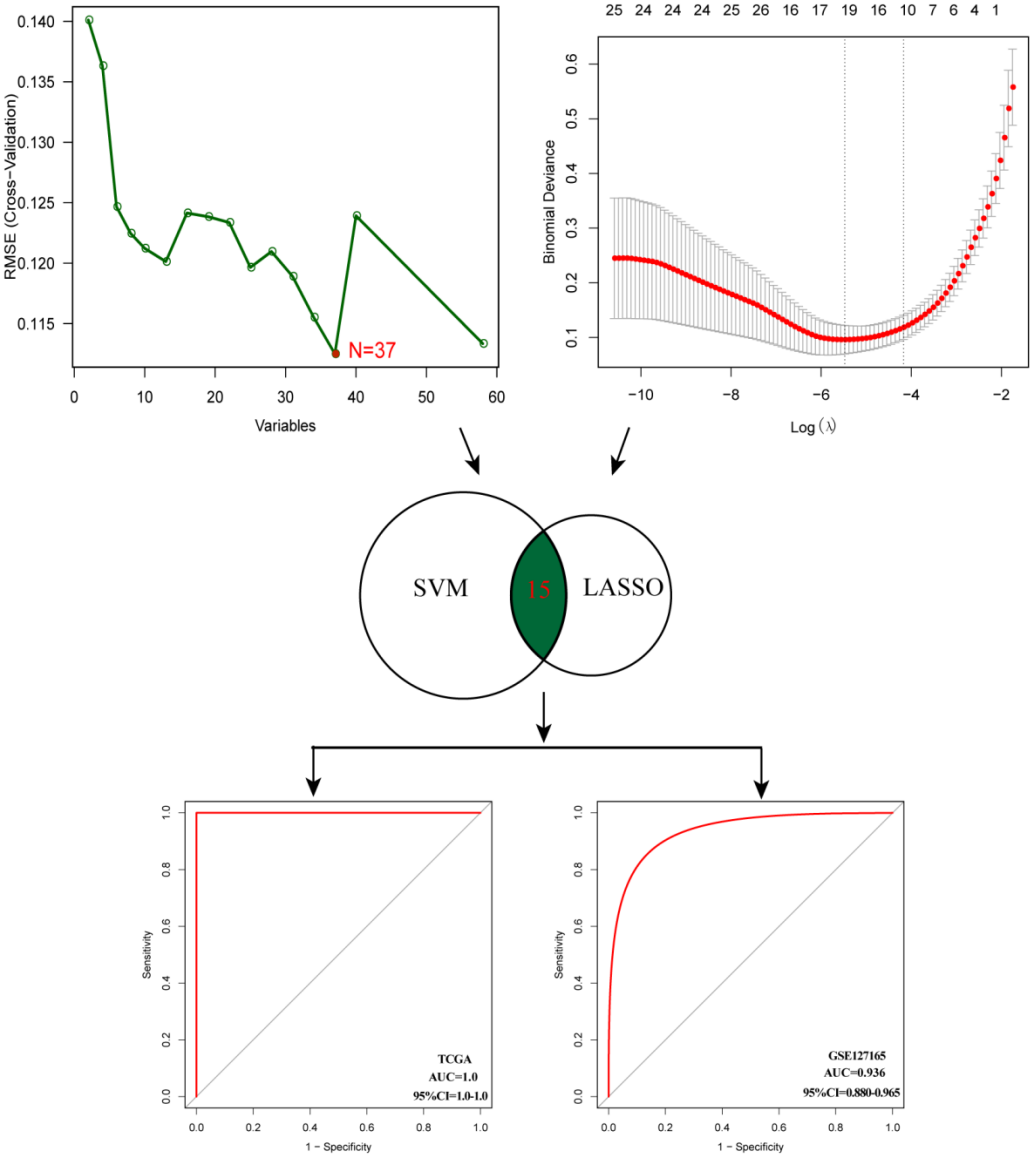
**Establishment of multigene diagnostic signature.** A total of 15 genes have been selected through SVM and LASSO analysis, and the AUC of the ROC curve is 0.997 (TCGA) and 0.978 (GSE127165).

After evaluating the prognostic value, we performed gene selection for diagnosis. 37 glycosyltransferase-related genes were extracted by SVM and 18 genes were screened by LASSO analysis, and after intersecting the two algorithms, we obtained 15 genes with diagnostic value (Supplementary Table 2). A diagnostic model was constructed based on the corresponding coefficients. In the TCGA cohort, the AUC value of the ROC curve was 0.997 (95CI%: 0.948–0.999), which suggested excellent diagnostic efficiency of the model (Figure 6). In addition, the above diagnostic model was also verified in GSE127165 (AUC:0.978, 95CI%: 0.947–0.991).

### B4GALT3 promotes tumor progression *in vitro*

**Figure 7:**
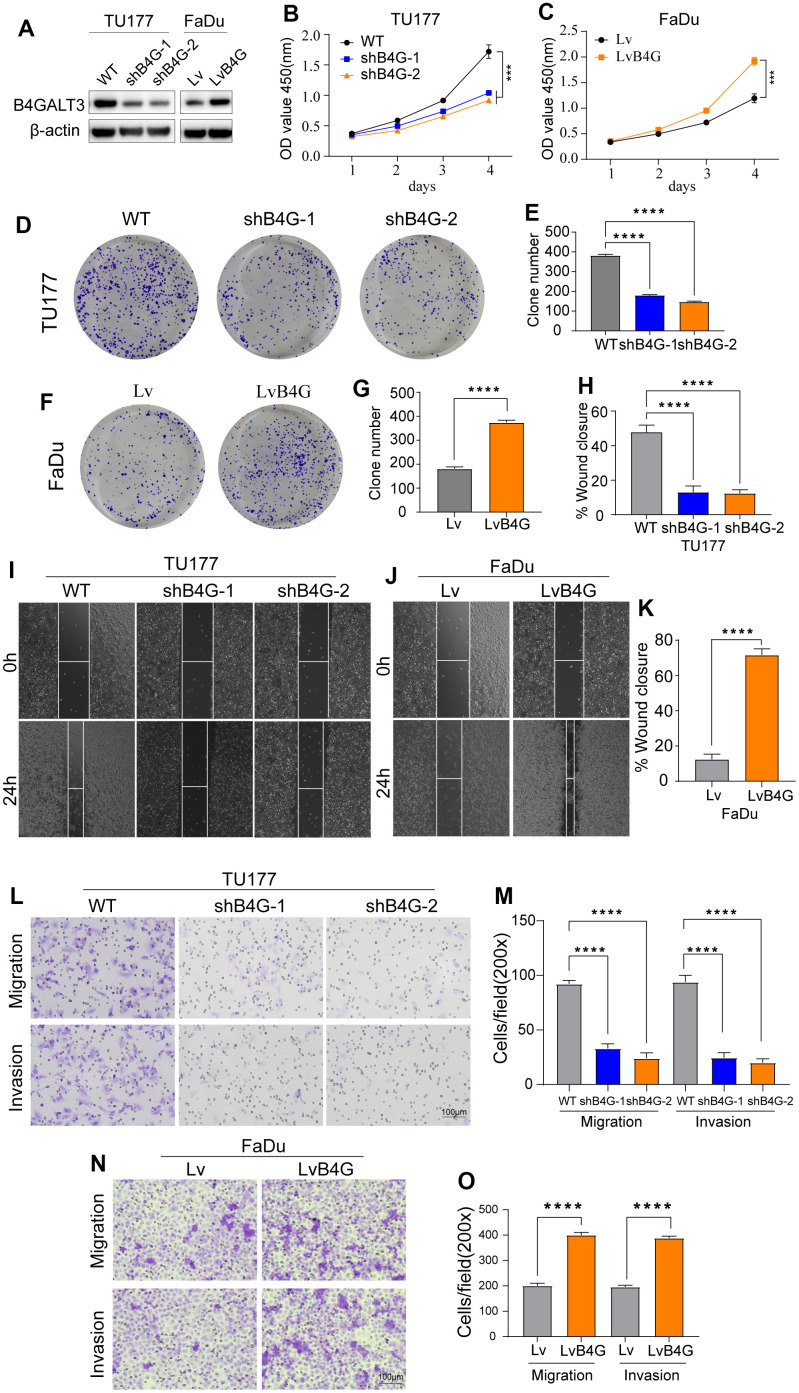
**B4GALT3 promotes the proliferation and migration of HNSCC cells.** (**A**) The expression of B4GALT3 has been assessed by western blotting. (**B**, **C**) CCK-8 assay has been performed to evaluate the growth rates of the indicated cells. The proliferative and migratory abilities of the indicated cells have been measured by colony formation (**D**–**G**) and wound healing assays (**H**–**K**), respectively. (**L**–**O**) Cell migration and invasion abilities of the indicated cells have been measured using Transwell assays.

To elucidate the biological functions of B4GALT3 in HNSCC, we created a stable TU177 cell line with a B4GALT3 knockdown. In contrast, we ectopically overexpressed the B4GALT3 gene in the FaDu cells. Western blotting was performed to assess the effectiveness of B4GALT3 knockdown and overexpression in these cell lines (Figure 7A). CCK-8 and colony formation assays revealed a notable decrease in the cell viability and colony-forming ability of TU177 cells upon knockdown of B4GALT3 (Figure 7B, 7D, 7E). Conversely, the overexpression of B4GALT3 in FaDu cells stimulated cell proliferation and enhanced colony formation (Figure 7C, 7F, 7G). Subsequently, we investigated whether B4GALT3 plays a role in HNSCC cell motility. Wound healing and Transwell assays revealed a significant enhancement in cell migration and invasion following B4GALT3 overexpression, whereas these migratory and invasive properties decreased upon B4GALT3 knockdown (Figure 7H–7O).

## DISCUSSION

Owing to the high molecular heterogeneity and anatomical location invisibility, early diagnosis and individualized treatment of patients with HNSCC have been great challenges [3]. With the rapid development of bioinformatics, several studies focus on finding key genes or biomarkers for assessing tumor prognosis and guiding treatment based on bioinformatics, and certain milestones have been achieved [25, 26]. However, previous bioinformatics studies were either limited to a single database or only focused on tumor outcomes, resulting in a lack of effective biomarkers that could simultaneously predict the diagnosis and prognosis of HNSCC. Many studies have begun to focus on the relationship between glycosyltransferases and their expression in tumors [27, 28]. However, there are few reports on the relationship between glycosyltransferases and HNSCC development. Therefore, we attempted to investigate the relevance of glycosyltransferase genes in the diagnosis and prognosis of HNSCC using bioinformatics technology and initially explored and established new biomarkers for the early diagnosis and prognosis prediction of HNSCC.

In the present study, we first identified five glycosyltransferase-related DEGs in the TCGA cohort using differential expression analysis and Cox regression and built a prognostic signature based on their respective correlation coefficients. Based on the median risk score, patients with HNSCC were classified into high- and low-risk subgroups. A comparison of the relationship between OS and the subgroups revealed that the OS of the low-risk group was longer. In addition, independent analyses suggested that the prognostic model was an independent prognostic factor for HNSCC. The validation process using the GSE41613 cohort confirmed the reliability of the results.

The prognostic glycosyltransferase-related gene signature comprised five genes: B4GALT3, PYGL, GALNT14, FUT2, and GALNT16. B4GALT3 is an enzyme in charge of the production of poly-N-acetyllactosamine and plays a crucial role in the occurrence, development, and metastasis of cancers. We knocked down and overexpressed B4GALT3 in both HNSCC cell lines. Depletion of B4GALT3 attenuated cell proliferation, migration, and invasion, whereas overexpression of B4GALT3 had the opposite effect. The B4GALT3 gene is upregulated in glioblastoma and cervical cancer and is considered to be related to tumor cell proliferation and invasion, suggesting a poor prognosis [29, 30]. Similar to B4GALT3, PYGL is upregulated in multiple tumors. Its expression level is positively correlated with glioma malignancy, and its high expression is an independent predictor of poor prognosis in glioma patients [31]. In addition, fucosyltransferase2 (FUT2) overexpression enhances cell migration and invasion *in vitro* and metastasis of breast cancer *in vivo* and may be used as a therapeutic target for breast cancer [32]. Both GALNT14 and 16 are members of the N-acetylgalactosaminyltransferase family. In breast cancer, GALNT14 modulates tumor multidrug resistance and promotes cancer invasion by altering cell proliferation, motility, and EMT gene expression levels [33, 34], while gene polymorphisms of GALNT16 are strongly associated with cancer susceptibility [35]. In summary, our findings demonstrated that these genes can serve as potential biomarkers for predicting the outcome of HNSCC.

Next, to gain further correlations between the prognostic model and the tumor microenvironment, gene mutations, and immune cell infiltration were compared between the two subgroups. We found that the TP53 mutation rate was the highest in both groups, and the high-risk group was higher than the low-risk group. As the most common single genetic event in cancer, TP53 mutation is related to poorer prognosis in several malignant tumors, which aligns with our findings [36, 37]. In addition, resting memory CD4 T cells, resting NK cells, M0 macrophages, and activated dendritic cells were more enriched in the high-risk subgroup, while naive B cells, plasma cells, CD8 T cells, and resting mast cells were more common in the low-risk subgroup. Previous studies have revealed that a large infiltration of T cells, particularly CD8 T cells, is generally associated with a favorable outcome for the tumor, while elevated levels of M0 macrophages are linked to a less favorable prognosis [38–40]. Some genes that constitute the prognostic model have been reported to be related to the tumor microenvironment. For example, B4GALT3 knockout mice demonstrated inhibited growth of highly immunogenic tumors accompanied by a significant elevation in the infiltration of CD8 T cells within the tumor microenvironment [41]. The number of macrophages significantly decreased in 231-LM2 cells when GALNT14 expression was suppressed, whereas it markedly increased upon GALNT14 overexpression [42].

ICI therapy has proven to be a useful treatment for carcinoma [43–45], and we observed that the risk score was negatively correlated with the immune checkpoints CTLA4, ICOS, and PDCD1, suggesting that the sensitivity to immunotherapy may differ among subgroups. The TIDE algorithm can effectively distinguish tumor immune escape and predict the efficacy of ICI therapy. In general, a higher TIDE score indicated an unfavorable response to immunotherapy [46]. In our study, the TIDE scores were lower in the high-risk group, suggesting lower levels of immune escape and higher ICI treatment efficiency in these samples. Notably, the complex interactions between cancer cells and the tumor microenvironment are closely related to the development of tumorigenesis and immunotherapy efficacy. Clarifying the tumor microenvironment of all patients with HNSCC can help us recognize which individuals are more likely to benefit from immunotherapy to develop individualized treatment strategies.

This study has certain limitations. First, the study was primarily conducted in public databases, and our outcomes were verified only in GEO datasets; further validation in additional clinical cases is required. Second, the genes included in this study were limited to glycosyltransferases, and the tumor immune microenvironment was complex, restricting the effectiveness of the prognostic model. However, these issues did not affect the results of this study.

## CONCLUSIONS

In summary, we performed the first integrated analysis of glycosyltransferase-related genes in HNSCC and discussed their significance in diagnosis and prognosis. These results are conducive to future prospective experimental studies. More importantly, we established two new biomarkers that could provide clinicians with diagnostic, prognostic, and treatment guidance for patients with HNSCC.

## Supplementary Material

Supplementary Figure 1

Supplementary Table 1

Supplementary Table 2

## References

[1] Siegel RL, Miller KD, Jemal A. Cancer statistics, 2020. CA Cancer J Clin. 2020; 70:7–30. 10.3322/caac.21590 31912902

[2] Ferlay J, Colombet M, Soerjomataram I, Mathers C, Parkin DM, Piñeros M, Znaor A, Bray F. Estimating the global cancer incidence and mortality in 2018: GLOBOCAN sources and methods. Int J Cancer. 2019; 144:1941–53. 10.1002/ijc.31937 30350310

[3] Chow LQ. Head and Neck Cancer. N Engl J Med. 2020; 382:60–72. 10.1056/NEJMra1715715 31893516

[4] Pinho SS, Reis CA. Glycosylation in cancer: mechanisms and clinical implications. Nat Rev Cancer. 2015; 15:540–55. 10.1038/nrc3982 26289314

[5] Munkley J, Elliott DJ. Hallmarks of glycosylation in cancer. Oncotarget. 2016; 7:35478–89. 10.18632/oncotarget.8155 27007155 PMC5085245

[6] Meany DL, Chan DW. Aberrant glycosylation associated with enzymes as cancer biomarkers. Clin Proteomics. 2011; 8:7. 10.1186/1559-0275-8-7 21906357 PMC3170274

[7] Ohtsubo K, Marth JD. Glycosylation in cellular mechanisms of health and disease. Cell. 2006; 126:855–67. 10.1016/j.cell.2006.08.019 16959566

[8] Wang W, Hu T, Frantom PA, Zheng T, Gerwe B, Del Amo DS, Garret S, Seidel RD 3rd, Wu P. Chemoenzymatic synthesis of GDP-L-fucose and the Lewis X glycan derivatives. Proc Natl Acad Sci USA. 2009; 106:16096–101. 10.1073/pnas.0908248106 19805264 PMC2752511

[9] Rodrigues JG, Balmaña M, Macedo JA, Poças J, Fernandes Â, de-Freitas-Junior JC, Pinho SS, Gomes J, Magalhães A, Gomes C, Mereiter S, Reis CA. Glycosylation in cancer: Selected roles in tumour progression, immune modulation and metastasis. Cell Immunol. 2018; 333:46–57. 10.1016/j.cellimm.2018.03.007 29576316

[10] Chandler KB, Costello CE, Rahimi N. Glycosylation in the Tumor Microenvironment: Implications for Tumor Angiogenesis and Metastasis. Cells. 2019; 8:544. 10.3390/cells8060544 31195728 PMC6627046

[11] Sun Y, Sun C, Zhang E. Expression of Serum Sialic Acid, Early Antigen-IgA, and Viral Capsid Antigen-IgA in Nasopharynx Cancer Patients: The Diagnostic Implication of Combined Assays. Med Sci Monit. 2015; 21:4068–73. 10.12659/msm.894951 26709095 PMC4699620

[12] Straka MB, Wagner RL, Johnson JT, Kachman KK, Eibling DE. The lack of utility of a tumor marker panel in head and neck carcinoma. Squamous cell carcinoma antigen, carcinoembryonic antigen, lipid-associated sialic acid, and CA-125. Arch Otolaryngol Head Neck Surg. 1992; 118:802–5. 10.1001/archotol.1992.01880080024007 1642830

[13] Bronikowska I, Świętochowska E, Oleksiak M, Czecior E. Sialic acids in squamous cell carcinoma of the head and neck. Postepy Hig Med Dosw (Online). 2016; 70:1300–8. 10.5604/17322693.1227410 28100840

[14] Bergler W, Stanek A, Riedel F, Petroianu G, Hörmann K. Role of sialoglycan structures for the function of the epidermal growth factor receptor and the *in vitro* proliferation of head and neck cancer. Eur Arch Otorhinolaryngol. 1998; 255:414–9. 10.1007/s004050050089 9801861

[15] Wilson JA, Rogers MJ, Hawkins RA, Gilmour HM, Maran AG. Epidermal growth factor receptors and oestrogen receptors in the head and neck. Clin Otolaryngol Allied Sci. 1993; 18:66–8. 10.1111/j.1365-2273.1993.tb00813.x 8448896

[16] Singhal A, Hakomori S. Molecular changes in carbohydrate antigens associated with cancer. Bioessays. 1990; 12:223–30. 10.1002/bies.950120506 1695095

[17] Huang TQ, Bi YN, Cui Z, Guan JP, Huang YC. MUC1 confers radioresistance in head and neck squamous cell carcinoma (HNSCC) cells. Bioengineered. 2020; 11:769–78. 10.1080/21655979.2020.1791590 32662743 PMC8291802

[18] Panier S, Boulton SJ. Double-strand break repair: 53BP1 comes into focus. Nat Rev Mol Cell Biol. 2014; 15:7–18. 10.1038/nrm3719 24326623

[19] Mohamed Abd-El-Halim Y, El Kaoutari A, Silvy F, Rubis M, Bigonnet M, Roques J, Cros J, Nicolle R, Iovanna J, Dusetti N, Mas E. A glycosyltransferase gene signature to detect pancreatic ductal adenocarcinoma patients with poor prognosis. EBioMedicine. 2021; 71:103541. 10.1016/j.ebiom.2021.103541 34425307 PMC8379629

[20] He Q, Yang J, Jin Y. Immune infiltration and clinical significance analyses of the coagulation-related genes in hepatocellular carcinoma. Brief Bioinform. 2022; 23:bbac291. 10.1093/bib/bbac291 35849048

[21] Chen B, Khodadoust MS, Liu CL, Newman AM, Alizadeh AA. Profiling Tumor Infiltrating Immune Cells with CIBERSORT. Methods Mol Biol. 2018; 1711:243–59. 10.1007/978-1-4939-7493-1_12 29344893 PMC5895181

[22] Liu Z, Mi M, Li X, Zheng X, Wu G, Zhang L. A lncRNA prognostic signature associated with immune infiltration and tumour mutation burden in breast cancer. J Cell Mol Med. 2020; 24:12444–56. 10.1111/jcmm.15762 32967061 PMC7687003

[23] Gao J, Kwan PW, Shi D. Sparse kernel learning with LASSO and Bayesian inference algorithm. Neural Netw. 2010; 23:257–64. 10.1016/j.neunet.2009.07.001 19604671

[24] Li D, Sun A, Zhang L, Ding Z, Yi F, Yang X, Wang Z, Chen X, Liu W, Liu S, Shen H, Miao M, Zhang L, et al. Elevated ITGA5 facilitates hyperactivated mTORC1-mediated progression of laryngeal squamous cell carcinoma via upregulation of EFNB2. Theranostics. 2022; 12:7431–49. 10.7150/thno.76232 36438491 PMC9691358

[25] Wu ZH, Yue JX, Zhou T, Xiao HJ. Integrated analysis of the prognostic values of RNA-binding proteins in head and neck squamous cell carcinoma. Biofactors. 2021; 47:478–88. 10.1002/biof.1722 33651487

[26] Jiang H, Ma B, Xu W, Luo Y, Wang X, Wen S, Liao T, Lu Z, Yang S, Wang Y. A Novel Three-lncRNA Signature Predicts the Overall Survival of HNSCC Patients. Ann Surg Oncol. 2021; 28:3396–406. 10.1245/s10434-020-09210-1 33095358

[27] Kuzmanov U, Kosanam H, Diamandis EP. The sweet and sour of serological glycoprotein tumor biomarker quantification. BMC Med. 2013; 11:31. 10.1186/1741-7015-11-31 23390961 PMC3751898

[28] Dube DH, Bertozzi CR. Glycans in cancer and inflammation--potential for therapeutics and diagnostics. Nat Rev Drug Discov. 2005; 4:477–88. 10.1038/nrd1751 15931257

[29] Wu T, Li Y, Chen B. B4GALT3 promotes cell proliferation and invasion in glioblastoma. Neurol Res. 2020; 42:463–70. 10.1080/01616412.2020.1740465 32202233

[30] Sun Y, Yang X, Liu M, Tang H. B4GALT3 up-regulation by miR-27a contributes to the oncogenic activity in human cervical cancer cells. Cancer Lett. 2016; 375:284–92. 10.1016/j.canlet.2016.03.016 26987623

[31] Zhao CY, Hua CH, Li CH, Zheng RZ, Li XY. High PYGL Expression Predicts Poor Prognosis in Human Gliomas. Front Neurol. 2021; 12:652931. 10.3389/fneur.2021.652931 34177761 PMC8225935

[32] Lai TY, Chen IJ, Lin RJ, Liao GS, Yeo HL, Ho CL, Wu JC, Chang NC, Lee AC, Yu AL. Fucosyltransferase 1 and 2 play pivotal roles in breast cancer cells. Cell Death Discov. 2019; 5:74. 10.1038/s41420-019-0145-y 30854233 PMC6403244

[33] Shan J, Liu Y, Wang Y, Li Y, Yu X, Wu C. GALNT14 Involves the Regulation of Multidrug Resistance in Breast Cancer Cells. Transl Oncol. 2018; 11:786–93. 10.1016/j.tranon.2018.04.003 29702465 PMC6058006

[34] Huanna T, Tao Z, Xiangfei W, Longfei A, Yuanyuan X, Jianhua W, Cuifang Z, Manjing J, Wenjing C, Shaochuan Q, Feifei X, Naikang L, Jinchao Z, Chen W. GALNT14 mediates tumor invasion and migration in breast cancer cell MCF-7. Mol Carcinog. 2015; 54:1159–71. 10.1002/mc.22186 24962947

[35] Wu H, He G, Song T, Zhang Y, Chen X, Chen H, Xiong W, Sun C, Zhao C, Chen Y. Evaluation of GALNT16 polymorphisms to breast cancer risk in Chinese population. Mol Genet Genomic Med. 2019; 7:e848. 10.1002/mgg3.848 31286696 PMC6687646

[36] Vousden KH, Prives C. P53 and prognosis: new insights and further complexity. Cell. 2005; 120:7–10. 10.1016/j.cell.2004.12.027 15652475

[37] Poeta ML, Manola J, Goldwasser MA, Forastiere A, Benoit N, Califano JA, Ridge JA, Goodwin J, Kenady D, Saunders J, Westra W, Sidransky D, Koch WM. TP53 mutations and survival in squamous-cell carcinoma of the head and neck. N Engl J Med. 2007; 357:2552–61. 10.1056/NEJMoa073770 18094376 PMC2263014

[38] Ali HR, Chlon L, Pharoah PD, Markowetz F, Caldas C. Patterns of Immune Infiltration in Breast Cancer and Their Clinical Implications: A Gene-Expression-Based Retrospective Study. PLoS Med. 2016; 13:e1002194. 10.1371/journal.pmed.1002194 27959923 PMC5154505

[39] Cai Z, Chen J, Yu Z, Li H, Liu Z, Deng D, Liu J, Chen C, Zhang C, Ou Z, Chen M, Hu J, Zu X. BCAT2 Shapes a Noninflamed Tumor Microenvironment and Induces Resistance to Anti-PD-1/PD-L1 Immunotherapy by Negatively Regulating Proinflammatory Chemokines and Anticancer Immunity. Adv Sci (Weinh). 2023; 10:e2207155. 10.1002/advs.202207155 36642843 PMC10015882

[40] Yang S, Wu Y, Deng Y, Zhou L, Yang P, Zheng Y, Zhang D, Zhai Z, Li N, Hao Q, Song D, Kang H, Dai Z. Identification of a prognostic immune signature for cervical cancer to predict survival and response to immune checkpoint inhibitors. Oncoimmunology. 2019; 8:e1659094. 10.1080/2162402X.2019.1659094 31741756 PMC6844304

[41] Wei H, Naruse C, Takakura D, Sugihara K, Pan X, Ikeda A, Kawasaki N, Asano M. Beta-1,4-galactosyltransferase-3 deficiency suppresses the growth of immunogenic tumors in mice. Front Immunol. 2023; 14:1272537. 10.3389/fimmu.2023.1272537 37901252 PMC10600447

[42] Song KH, Park MS, Nandu TS, Gadad S, Kim SC, Kim MY. GALNT14 promotes lung-specific breast cancer metastasis by modulating self-renewal and interaction with the lung microenvironment. Nat Commun. 2016; 7:13796. 10.1038/ncomms13796 27982029 PMC5171903

[43] Li H, Chen J, Li Z, Chen M, Ou Z, Mo M, Wang R, Tong S, Liu P, Cai Z, Zhang C, Liu Z, Deng D, et al. S100A5 Attenuates Efficiency of Anti-PD-L1/PD-1 Immunotherapy by Inhibiting CD8+ T Cell-Mediated Anti-Cancer Immunity in Bladder Carcinoma. Adv Sci (Weinh). 2023; 10:e2300110. 10.1002/advs.202300110 37414584 PMC10477882

[44] Hu J, Chen J, Ou Z, Chen H, Liu Z, Chen M, Zhang R, Yu A, Cao R, Zhang E, Guo X, Peng B, Deng D, et al. Neoadjuvant immunotherapy, chemotherapy, and combination therapy in muscle-invasive bladder cancer: A multi-center real-world retrospective study. Cell Rep Med. 2022; 3:100785. 10.1016/j.xcrm.2022.100785 36265483 PMC9729796

[45] Bellmunt J, de Wit R, Vaughn DJ, Fradet Y, Lee JL, Fong L, Vogelzang NJ, Climent MA, Petrylak DP, Choueiri TK, Necchi A, Gerritsen W, Gurney H, et al, and KEYNOTE-045 Investigators. Pembrolizumab as Second-Line Therapy for Advanced Urothelial Carcinoma. N Engl J Med. 2017; 376:1015–26. 10.1056/NEJMoa1613683 28212060 PMC5635424

[46] Jiang P, Gu S, Pan D, Fu J, Sahu A, Hu X, Li Z, Traugh N, Bu X, Li B, Liu J, Freeman GJ, Brown MA, et al. Signatures of T cell dysfunction and exclusion predict cancer immunotherapy response. Nat Med. 2018; 24:1550–8. 10.1038/s41591-018-0136-1 30127393 PMC6487502

