# Supplementary material for: Development and validation of glycosyltransferase related-gene for the diagnosis and prognosis of head and neck squamous cell carcinoma
Source: Aging (Albany NY). 2024 Jan 19;16(2):1750–66. doi: 10.18632/aging.205455 (PMC10866440; doi:10.18632/aging.205455)
Supplement: Supplementary Figure 1 [file aging-16-205455-s001.pdf]

SUPPLEMENTARY FIGURE

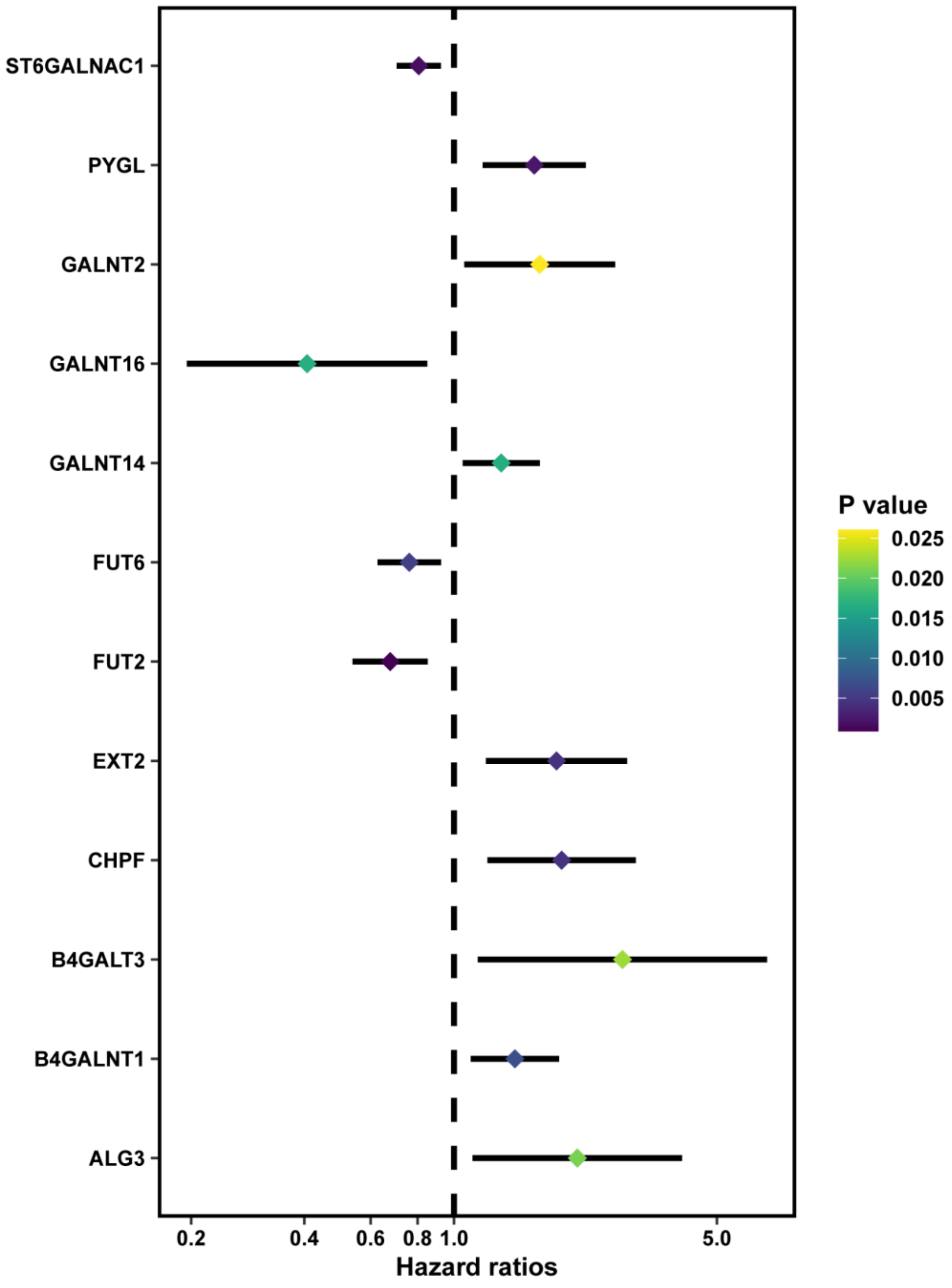

Supplementary Figure 1. The HR values of univariate Cox regression analysis.
