# Supplementary material for: Development and validation of glycosyltransferase related-gene for the diagnosis and prognosis of head and neck squamous cell carcinoma
Source: Aging (Albany NY). 2024 Jan 19;16(2):1750–66. doi: 10.18632/aging.205455 (PMC10866440; doi:10.18632/aging.205455)
Supplement: Supplementary Table 1 [file aging-16-205455-s002.docx]

**Supplementary Table 1. List of 169 glycosyltransferase related genes.**

| FUT1 |
| --- |
| FUT2 |
| FUT3 |
| FUT4 |
| FUT5 |
| FUT6 |
| FUT7 |
| FUT8 |
| FUT9 |
| FUT10 |
| FUT11 |
| POFUT1 |
| POFUT2 |
| ST3GAL1 |
| ST3GAL2 |
| ST3GAL3 |
| ST3GAL4 |
| ST3GAL5 |
| ST3GAL6 |
| ST6GALNAC1 |
| ST6GALNAC2 |
| ST6GALNAC3 |
| ST6GALNAC4 |
| ST6GALNAC5 |
| ST6GALNAC6 |
| ST6GAL1 |
| ST6GAL2 |
| ST8SIA1 |
| ST8SIA2 |
| ST8SIA3 |
| ST8SIA4 |
| ST8SIA5 |
| ST8SIA6 |
| GALNTL6 |
| GALNT1 |
| GALNT2 |
| GALNT3 |
| GALNT4 |
| GALNT5 |
| GALNT6 |
| GALNT7 |
| GALNT8 |
| GALNT9 |
| GALNT10 |
| GALNT11 |
| GALNT12 |
| GALNT13 |
| GALNT14 |
| GALNT15 |
| GALNT16 |
| GALNT18 |
| B3GALNT1 |
| B3GALNT2 |
| B3GALT1 |
| B3GALT2 |
| B3GALT4 |
| B3GALT5 |
| B3GALT6 |
| B3GNT2 |
| B3GNT3 |
| B3GNT4 |
| B3GNT5 |
| B3GNT6 |
| B3GNT7 |
| B3GNT8 |
| B3GNT9 |
| C1GALT1 |
| LFNG |
| MFNG |
| RFNG |
| MGAT1 |
| MGAT2 |
| MGAT3 |
| MGAT4A |
| MGAT4B |
| MGAT4C |
| MGAT5 |
| MGAT5B |
| B4GALNT1 |
| B4GALNT2 |
| B4GALNT3 |
| B4GALNT4 |
| B4GALT1 |
| B4GALT2 |
| B4GALT3 |
| B4GALT4 |
| B4GALT5 |
| B4GALT6 |
| B4GALT7 |
| CHPF |
| CHPF2 |
| CHSY1 |
| CHSY3 |
| CSGALNACT1 |
| CSGALNACT2 |
| A4GALT |
| A4GNT |
| GCNT1 |
| GCNT2 |
| GCNT3 |
| GCNT4 |
| GCNT7 |
| XYLT1 |
| XYLT2 |
| GLT8D1 |
| GLT8D2 |
| GXYLT1 |
| GXYLT2 |
| GYG1 |
| GYG2 |
| XXYLT1 |
| EOGT |
| OGT |
| POMGNT1 |
| POMGNT2 |
| ABO |
| GBGT1 |
| ALG1 |
| ALG1L |
| ALG1L2 |
| ALG2 |
| ALG11 |
| GLT1D1 |
| GTDC1 |
| GYS1 |
| GYS2 |
| PIGA |
| ALG10 |
| ALG10B |
| ALG6 |
| ALG8 |
| ALG3 |
| ALG9 |
| ALG12 |
| PIGB |
| PIGM |
| PIGV |
| PIGZ |
| POMT1 |
| POMT2 |
| ALG5 |
| B3GNTL1 |
| DPM1 |
| HAS1 |
| HAS2 |
| HAS3 |
| EXTL1 |
| EXTL2 |
| EXTL3 |
| EXT1 |
| EXT2 |
| PYGB |
| PYGL |
| PYGM |
| COLGALT1 |
| COLGALT2 |
| UGGT1 |
| UGGT2 |
| POGLUT2 |
| POGLUT3 |
| POGLUT1 |
| STT3A |
| STT3B |
| ALG13 |
| ALG14 |
| UGCG |
| B3GAT1 |
| B3GAT2 |
| B3GAT3 |
