# Supplementary material for: Development and validation of glycosyltransferase related-gene for the diagnosis and prognosis of head and neck squamous cell carcinoma
Source: Aging (Albany NY). 2024 Jan 19;16(2):1750–66. doi: 10.18632/aging.205455 (PMC10866440; doi:10.18632/aging.205455)
Supplement: Supplementary Table 2 [file aging-16-205455-s003.pdf]

SUPPLEMENTARY TABLE

Supplementary Table 2. List of genes used to construct the diagnostic model.

SVM	LASSO	interGenes
COLGALT1	COLGALT1	COLGALT1
ALG3	GYS2	GYS2
EXT1	ALG3	ALG3
ALG1L	EXTL1	EXTL1
GALNT16	ALG1L	ALG1L
GYS2	GALNT16	GALNT16
B3GNT4	CSGALNACT2	B3GNT6
FUT6	B3GNT6	EXTL3
B3GAT3	EXTL3	B3GNT9
ABO	B3GNT9	XXYLT1
B4GALNT1	XXYLT1	B3GNT4
XYLT2	B3GNT4	FUT2
CHPF2	GALNT10	B4GALNT1
EXTL1	FUT2	GALNT5
B4GALNT3	B4GALNT1	B3GNT8
FUT2	GALNT5	
GCNT3	B3GNT7	
PYGM	B3GNT8	
GALNT5		
B4GALT2		
XXYLT1		
EXTL3		
GALNT2		
PYGL		
LFNG		
B3GNT6		
GALNT6		
POFUT1		
GALNT12		
ST6GALNAC1		
B4GALNT4		
GALNT18		
ST6GALNAC2		
B3GNT8		
B3GNT9		
B4GALT1		
EXTL2		
